# Assessment of total mercury content in fish muscle tissue from the middle basin of the Pastaza River, Ecuador

**DOI:** 10.1371/journal.pone.0310688

**Published:** 2024-12-18

**Authors:** Fernanda Paz-Suconota, Lenys Fernández, Natalia Carpintero-Salvador, Melany Ruiz-Urigüen, Stefan Alexander Brück, Fidel Ernesto Rodríguez Galarza, Ricardo Burgos-Morán, Patricio J. Espinoza-Montero

**Affiliations:** 1 Escuela de Ciencias Químicas, Pontificia Universidad Católica del Ecuador, Quito, Ecuador; 2 Facultad de Ciencias Biológicas, Universidad Central del Ecuador, Quito, Ecuador; 3 Core Lab de Ciencias Ambientales, Universidad San Francisco de Quito, Quito, Ecuador; 4 Ingeniería Ambiental, Colegio Politécnico, Universidad San Francisco de Quito, Quito, Ecuador; 5 Instituto de Estudios Amazónicos e Insulares, Universidad Central del Ecuador, Quito, Ecuador; 6 Estación Científica Amazónica Juri Juri Kawsay, Universidad Central del Ecuador, Quito, Ecuador; 7 Departamento de Ciencias de la Tierra, Universidad Estatal Amazónica, Pastaza, Ecuador; BOKU: Universitat fur Bodenkultur Wien, AUSTRIA

## Abstract

One of the most toxic metals is mercury, which exhibits high toxicity during short exposure periods. This study aimed to determine the concentration of total mercury (THg) in fish muscle tissue from various species captured from two locations in the middle basin of the Pastaza River in Ecuador, which the locals use in their weekly diet. The individuals captured belong to the following families: Loricariidae, Cetopsidae, Heptapteridae, Cichlidae, Parodontidae, Characidae, Prochilodontidae, Curimatida, Siluriformes, Cichliformes, Characiformes, Erythrinidae and Curimatidae. Carnivorous species *Charax* sp. and *Cetopsis plumbea*, had THg concentrations were 0.241± 0.018 and 0.116 ± 0.045 mg kg^-1^, respectively. Herbivorous species *Hypostomus* sp. had a lower of THg. Carnivorous species *Hoplias malabaricus* exhibited THg concentration of 0.160±0.033 and 0.020±0.007 mg kg^-1^ for the omnivorous species *Creagrutus* sp. Carnivorous species accumulated higher levels of Hg compared to non-carnivorous species. Concentration of total mercury in the collected fish did not exceed the maximum permissible limits set by legislative standards (Ecuadorian Institute of Standardization (INEN as per the acronym in Spanish), FAO/WHO and European Commission); and the objective hazard quotient was less than 1, indicating that the consumption of this fish may pose no risk to human health. Considering the mean concentrations of methylmercury, for all the fish species analysed, the results for daily consumption rate were between 6–199 g and 29–928 g for children and adults, respectively.

## Introduction

Metals and metalloids naturally exist in the environment, including water resources, however, when in excess, they can become pollutants; altering the physical, chemical, and biological characteristics of the environment, posing risks to the survival of aquatic biota, food security, and human health [1–4].

In freshwater environments, the presence of metals and metalloids is mainly due to deforestation, household wastewater discharge, agricultural activities and other industries [5–8], however, in recent years, the increase in mining activities for Au extraction [9, 10], and other minerals, could contribute to the release processes of mercury (Hg) in the ecosystems of Ecuador.

Metals and metalloids in rivers are able to bioaccumulate, a process involving water, sediment, and the food chains of the ecosystem’s resident organisms. This magnification substantially impacts local fish populations and is influenced by factors such as metal assimilation by fish tissues, duration of exposure, and environmental conditions including salinity, pH, water hardness, and temperature [11]. Metals and metalloids enter fish through two primary mechanisms: adsorption and absorption [12]. Adsorption takes place on tissue membranes, while absorption occurs through the ingestion of food and particulate matter present in the surrounding environment [13].

One of the most toxic metals is Hg [14], which exhibits high toxicity during short exposure periods [12], furthermore “Hg is potent neurotoxic agent, consumption of fish high on the trophic chain is a growing public health concern” [15, 16]. Mercury has the ability to accumulate in organisms, persist in the environment, and be dispersed over extensive distances [17]. Approximately 80% of total mercury (THg) in fish muscle is present in the form of methylmercury (MeHg), which is the most toxic chemical species of Hg [18]. Fish at higher trophic levels, particularly carnivorous and piscivorous species, accumulate the most metal content [19]. However, high levels of Hg have been reported in detritivorous species, associated with the availability of the metal in water sediments [20]. Fish species accumulate the contaminant in their tissues over their life cycle, with a correlation observed between age—determined by the size or weight of the individual fish—and Hg levels. In Amazonian species, four bioaccumulation patterns have been identified: positive linear, positive stepped, neutral, and negative stepped, with certain cases showing negative linear patterns, as well [21, 22].

Environmental pollution in the Ecuadorian Amazon has increased due to the region’s population growth. By 2010, the estimated population totalled 500,000 inhabitants, with 61% lacking basic public infrastructure such as sewage systems and wastewater treatment facilities [23]. As a result, the disposal of resident-generated waste along the riverbanks is still common practice in Amazonian urban centers, leading to the accumulation of various pollutants, including toxic metals [24]. Furthermore, the industrializations process contributes to increase the emissions by fossil fuel burning [10], plus mining activities become on the whole the principal sources of Hg pollution in aquatic environments in the amazon region in Ecuador [9].

The Pastaza River basin is part of the Amazonian watershed in Ecuador. It originates at the foot of the Tungurahua volcano, formed by the confluence of the Patate and Chambo rivers. From there, it flows through forests and wetlands until reaching Peru, where it empties into the Marañón River [25]. Much of the territory within the Pastaza River basin is threatened by anthropogenic activities, particularly the construction of hydroelectric plants and agricultural expansion [25–27]. The Metzeras River is a micro-basin of the Pastaza River and considered the largest water tributary that crosses the urban parish of the Palora Canton [28]. Within this area, anthropogenic activities, including the disposal of agricultural waste containing pesticides and agrochemicals, sewage discharge, and effluents from small-scale industries, contribute to the contamination of the water system [29].

When pollution occurs in aquatic environments, fish are one of the main species affected [30]. This is important because freshwater fish are frequently consumed by residents of cities and areas adjacent to rivers as they are a vital source of protein and vitamins [31]. Understanding the transfer of Hg in food chains is crucial for clarifying the impacts of anthropogenic disturbance on aquatic environments; even more so in areas like the Amazon region, where fish are a main component of the communities daily diet [21, 32].

In the present study, THg was quantified in the dorsal muscle of fish species inhabiting the middle basin of the Metzeras River (Site 1) and the Pastaza River (Site 2), located in the Amazon region of Ecuador. Fish species with different feeding habits were selected and classified from two distinct zones of the river, each characterized by varying levels of disturbance from agricultural activities.

## Material and methods

### Area of study

The middle basin of the Pastaza River (S1 Fig) includes three provinces: Tungurahua (Baños de Agua Santa Canton), Pastaza (Mera and Pastaza cantons), and Morona Santiago (Palora Canton) [27]. Sampling was carried out at two sites in the water basin in Palora Canton (Morona Santiago). Palora Canton is located in the Amazon region of Ecuador, in the north-western area of Morona Santiago Province. It is home to approximately 7,553 inhabitants, covering an area of 1,455.64 km^2^. The canton comprises five parishes: one urban (Palora or Metzeras) and four rural (Cumandá, 16 de Agosto, Sangay, and Arapicos) [33]. Thanks to its location, the Palora Canton boasts rich biodiversity of flora and fauna. Its terrain is predominantly flat, characterized by plains and sandy loam soils [29].

#### Site 1: Metzeras River

The Metzeras River is located in the urban parish (Palora/Metzeras) of Palora Canton in Morona Santiago Province. It lies in the pluvial bioclimatic region at an elevation of 920 masl. The predominant forest type in the canton is the Piedemonte evergreen forest, interspersed with intervened areas. It has an average annual temperature of 20.86°C, an annual precipitation ranging from 280 to 300 mm^3^, and a humid tropical microclimate [34]. Owing to anthropogenic activities, water systems are contaminated by agricultural waste, sewage discharge, and effluents from small-scale industries [29]. It is regarded as the largest water tributary traversing the urban parish, with 880 inhabitants settled along its banks, accounting for 25% of the urban population [28].

#### Site 2: Pastaza River at the Santa Inés community

The Pastaza River is estimated to be the third largest drainage basin in the country, with an area of 23,469.27 km^2^. Along its length, the river exhibits a rich diversity of flora and fauna, along with natural areas, agricultural lands, urban settlements, and indigenous communities [27]. In Palora, its waters flow to the northern part of the canton, within the 16 de Agosto Parish, characterized by flat terrain with loamy soils. The predominant forest type is the very humid pre-montane forest, found at altitudes from 600 to 2000 masl. It has an annual temperature of between 18 and 24°C and an annual precipitation between 2000 and 4000 mm^3^, indicating high rainfall [35, 36]. Over time, the Pastaza River has changed as a result of ecosystem deterioration. Within the parish, pollution of the water system is mainly associated with agriculture and solid waste discharge [36].

### Sampling

The study used an analytical, observational, and prospective approach. Samples of fish from the two sites were collected to determine the quality and state of the fish stocks. Due to its temporal nature, the study was cross-sectional, conducted over a specific period of time, described below.

The study population consisted of fish species from two sites in the middle basin of the Pastaza River in Morona Santiago Province, Ecuador. The sample comprised the muscle tissue of 40 specimens with different feeding habits (i.e., carnivores, detritivores, omnivores, and herbivores). The sampling was carried out at both sites on October 19–23, 2023. Individuals were collected using the cast-net fishing method, employing upstream throws at each site. The study was conducted along 1.3 km of the Metzeras River (Site 1) and 1 km of the Pastaza River (Site 2). Site 1 constituted an aquatic habitat approximately 7 m wide, characterized by a sandy and rocky bottom and moderate water transparency and flow. It was surrounded by a humid forest and pitahaya crops. In contrast, Site 2 had a width of approximately 20 m, with depths exceeding 2 m, sandy and rocky shores, low water transparency, and moderate water flow. It was surrounded by dense vegetation, including marginal plants. Sampling was carried out at night (19:00–22:30), with live captures. The collected fish were placed in a 20 L container with an oxygenator until they were transferred to the storage site. To euthanize the individuals, they were placed in a tray and sprayed with the anesthetic Roxicaine 10 times. Each individual was photographed with its respective label. Subsequently, morphometric parameters such as total weight, standard length (from the head to the beginning of the caudal fin), and total length (from the head to the end of the caudal fin) were measured [37]. Muscle samples weighing 6 grams were extracted from the dorsal area and placed in Ziplock bags labeled accordingly. They were then refrigerated until transportation to Quito, Ecuador. The remainder of each specimen was injected with 10% formalin, preserved in 75% alcohol in glass jars, and deposited at the National Biodiversity Institute of Ecuador (Instituto Nacional de Biodiversidad, INABIO). The taxonomic identification of each individual was conducted according to the literature [38–44].

### Mercury quantification

The muscle samples extracted from each individual were kept frozen at -20°C. They were then transferred to petri dishes and lyophilized using the Virtis Advantage Plus Lyophilizer for 34 hours. The lyophilized samples were weighed, grounded, sieved (N°35 mesh), and preserved in containers labeled accordingly for storage in a low-humidity environment until analysis. THg was quantified using a Milestone DMA-80 Direct Mercury Analyzer from the Core Lab, according to the following procedure: 40 mg of dried sample were placed on the analyzer’s niquel sample boat. It was then introduced into a catalysis tube, where two processes were initiated: first, it was dried and then thermally decomposed in an aerobic environment [45, 46]; second, halogens, nitrogen, and sulfur oxides were trapped and removed through continuous gas flow. Mercury vapor was then released and carried via compressed air to the amalgamator, where it was captured [47]. Subsequently, the amalgamator was heated to release Hg, which then passed through two absorbance cells with different measurement ranges (cell 1: 0.5–20 ng Hg; cell 2: 20–1000 ng Hg), where Hg was quantified. The atomic absorption spectrophotometer used a 254 nm Hg lamp [48].

All analysis were carried out in triplicate. Additionally, three blank samples were used at the beginning of each reading and after every 15 samples. The blank samples consisted of empty sample vials, which allowed for verification that the equipment was free from any contamination that could interfere with the sample readings [48]. The DORM-4 certified reference material (CRM) (fish protein) was used as a control at the beginning of the reading and after every 10 samples.

The accuracy of the methodology employed in the study was assessed by analyzing the DORM-4 CRM. The percentage of THg recovery was calculated based on the CRM value (412 ± 36 μg kg^-1^). The percent recovery from the five DORM-4 readings ranged from 90 to 96%, with a mean value of 92.74% and a coefficient of variation of 2.46% (S1 Table). The limits of detection (LDM) and quantification (LQM) were obtained from the nine blank readings, Eqs (1) and (2), respectively, with values of 1.14 μg kg^-1^ and 3.80 μg kg^-1^, respectively (S2 Table).


$$\text{LDM}=\frac{3.3{\left(1+\text{h}0\right)}^{\frac{1}{2}}\times s_{y}}{\text{b}}$$ (1)

LQM=3LDM (2)

Descriptive statistics, including mean, standard deviation, range, and recovery, were calculated using Microsoft Excel 2019 and BioEstat 5.3. The normality of the data distribution was assessed using the Shapiro–Wilk test. As the data did not follow a normal distribution, nonparametric tests were conducted. The Mann–Whitney U test was employed to compare and establish significant differences between the two study sites, while the Kruskal–Wallis test was used to determine significant differences across categories based on feeding habits. The relationship between THg concentration and individual length was assessed using the Pearson correlation coefficient. A significance level of *p* < 0.05 was set for all statistical tests.

### Human health risk assessment

As per the United States Environmental Protection Agency [49], to safeguard human health, THg should be regarded as MeHg in the whole fish sample. Consequently, for this study, the potential health risk was evaluated using THg concentrations as MeHg in the muscle tissue of fish.

The level of exposure (Ex) for MeHg was calculated using the Eq (3) [49]:

$$Ex=\frac{\text{Cx}\times \text{CR}}{\text{BW}}$$ (3)

Where Cx is the concentration of metal in the edible portion of the samples (mg·kg^-1^), CR is the fish ingestion rate per day (kg⋅d^-1^), 40 g for children and 80 g for adults, and BW is the average mean body weight (kg), 15 kg for children and 70 kg for adults.

The non-carcinogenic health risk assessment (Rx) through fish consumption was calculated by Eq (4) [50]:

$$Rx=\frac{Ex}{\text{RfD}}$$ (4)

Where Ex is the exposure to the pollutant (mg·kg^-1^·d^-1^), and RfD is the reference dose of MeHg (1x10^-4^ mg·kg^-1^·d^-1^) [50, 51]. A health risk is considered to exist if the calculated non-carcinogenic risk value exceeds 1; for values less than 1, there are no health risks.

The approximate grams of fish that can be consumed per day with an acceptable risk, the permissible daily consumption rate for health was calculated by Eq (5).


$$CRlim=\frac{RDF\;x\;BW}{C\text{x}}\times 1000$$ (5)

The Estimated Daily Intake (EDI) was calculated, Eq 1 [49], to approximate the daily Hg intake for a consumer of a specified body weight (adult or child) through the consumption of contaminated fish and Target hazard quotient (tHQ) by Eq (7).


$$\text{EDI}=\frac{\text{C}\times \text{FIR}\times \text{EFr}\times \text{ED}}{\text{RfD}\times \text{BW}\times \text{ATn}}$$ (6)

$$\text{tHQ}=\frac{\text{EDI}}{\text{RfD}}$$ (7)

Where C is the concentration of THg in fish (mg kg^- 1^ ww); FIR is the fish ingestion rate for children and adults of both sexes (0.057 kg week and 0.113 kg week, respectively); EFr is the exposure frequency, or the number of exposure events per year (ranging from 365 days year for individuals consuming fish seven times per week to 52 days year for those consuming fish once per week); ED is the exposure duration (70 years for adults and 6 years for children) [52–54], equivalent to the half-life; RfD is the specific oral reference dose (0.4 μg g^-1^ day^-1^) [55]; BW is body weight; and ATn is the average time of exposure to non-carcinogenic substances (EFr × ED) (days). For contaminated fish, the target hazard quotient (tHQ) utilizes the oral reference dose (RfD) to estimate the non-carcinogenic health risk for consumers from the intake of trace metals [56]; the RfD takes into account the daily exposure level deemed to be without significant health risks over a consumer’s lifetime [57]. A tHQ > 1 indicates a high non-carcinogenic adverse health risk from consuming fish, whereas a tHQ < 1 suggests no adverse health effects [54].

## Results and discussion

### Sampling

A total of 40 individuals were captured, 25 from Site 1, and 15 from Site 2. The individuals captured from Site 1, depicted in S3A Fig, belong to the following families: Loricariidae, Cetopsidae, Heptapteridae, Cichlidae, Parodontidae, Characidae, Prochilodontidae, and Curimatidae. These families are included in the orders Siluriformes, Cichliformes, and Characiformes. The individuals captured at Site 2, S3B Fig, belong to the families Loricariidae, Cichlidae, Erythrinidae, Curimatidae, Characidae, and Prochilodontidae from the orders Siluriformes, Cichliformes, and Characiformes. The results are summarized in Tables 1 and 2.

**Table 1:** Morphological data of the species collected in Site 1.

Order	Family	Species	n	Weight (g)	Length(cm)	Total length (cm)	Feeding habit
**Siluriformes**	Loricariidae	*Chaetostoma* sp.	2	64.5 ± 6.4	12.75 ± 0.4	16.0 ± 2.4	Herbivore
		*Hypostomus* sp.	1	112± 7.4	17.5± 02	24.0± 1.2	Herbivore
		*Cordylancistrus* sp.	3	45.3 ± 14.4	12.4 ± 0.9	15.4 ± 0.9	Herbivore
	Cetopsidae	*Cetopsis plumbea*	2	44.5 ± 2.1	13.8 ± 0.4	16.3 ± 1.1	Carnivorous
	Heptapteridae	*Pimelodella* sp.	3	24.7 ± 4.7	12.4 ± 1	15.9 ± 1	Omnivore
**Cichliformes**	Cichlidae	*Crenicichla anthurus*	3	78.0 ± 38.3	17.5 ± 3.3	20.8 ± 3.1	Carnivore
**Characiformes**	Parodontidae	*Parodon buckleyi*	3	42.3 ± 3.1	13.6 ± 0.6	16.4 ± 0.4	Omnivore
	Characidae	*Astyanax bimaculatus*	3	21.3 ± 4.0	9.7 ± 0.9	11.8 ± 1.1	Omnivore
		*Creagrutus* sp.	1	19.0± 2.4	10.0± 0.8	12.0± 3.1	Omnivore
		*Charax* sp.	1	26.0± 3.3	11.1± 1.3	13.5± 1.3	Carnivorous
	Prochilodontidae	*Prochilodus nigricans*	2	280.5 ± 23.3	24.5 ± 1.2	29.0 ± 1.9	Detritivore
	Curimatidae	*Steindachnerina* sp.	1	19.0± 16.4	9.3± 0.9	11.3± 0.2	Detritivore

**Table 2:** Morphological data of the species collected in Site 2.

Order	Family	Species	n	Weight (g)	Standard length (cm)	Total length (cm)	Eating habit
**Siluriformes**	Loricariidae	*Chaetostoma* sp.	3	60.3 ± 8.7	12.8 ± 0.3	16.3 ± 0.3	Herbivore
**Cichliformes**	Cichlidae	*Aequidens tetramerus*	3	79.3 ± 17.5	12.6 ± 1.4	17.1 ± 1.6	Omnivore
**Characiformes**	Erythrinidae	*Hoplias malabaricus*	2	106 ± 56.6	18.25 ± 3.2	23 ± 4.2	Carnivore
	Curimatidae	*Steindachnerina* sp.	3	18.7 ± 0.6	8.6 ± 0.2	11.2 ± 0.3	Detritivore
	Characidae	*Creagrutus* sp.	3	17 ± 2.0	9.3 ± 0.6	11.8 ± 0.5	Omnivore
	Prochilodontidae	*Prochilodus nigricans*	1	34.2± 2.3	25.5± 4.4	31.0 ± 3.2	Detritivore

### Mercury quantification

According to the standards from the Ecuadorian Institute of Standardization (INEN as per the acronym in Spanish), the Codex Alimentarius Commission of the Food and Agriculture Organization/World Health Organization (FAO/WHO), and the European Commission, the maximum permissible levels of Hg in fish meat vary between 0.5 and 1 mg kg^-1^, depending on the fish species [58–60].

**Fig 1:**
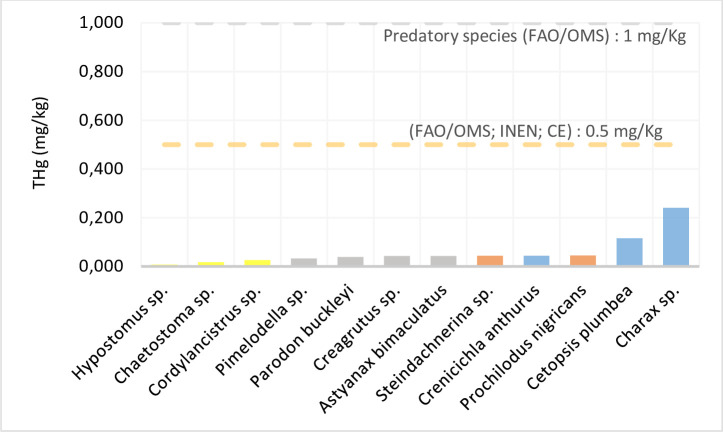
Total mercury concentration (mg kg^-1^) of the species collected in the Metzeras River.

Concentration of total mercury in muscle tissue of the species collected at Site 1 are shown in S3 Table. The highest THg content corresponded to *Charax* sp., followed by *Cetopsis plumbea*, with mean values of 0.241 and 0.116 ± 0.045 mg kg^-1^, respectively. *Hypostomus* sp. and *Chaetostoma* sp. contained lower levels, with mean values of 0.008 and 0.017 ± 0.002 mg kg^-1^, respectively. As presented in S3 Table, the Hg concentrations of the fish from Site 1 did not exceed the maximum limits allowed by the aforementioned organizations. The highest concentration of THg was found in carnivorous species and the lowest in herbivorous species. The results are summarized in Fig 1.

**Fig 2:**
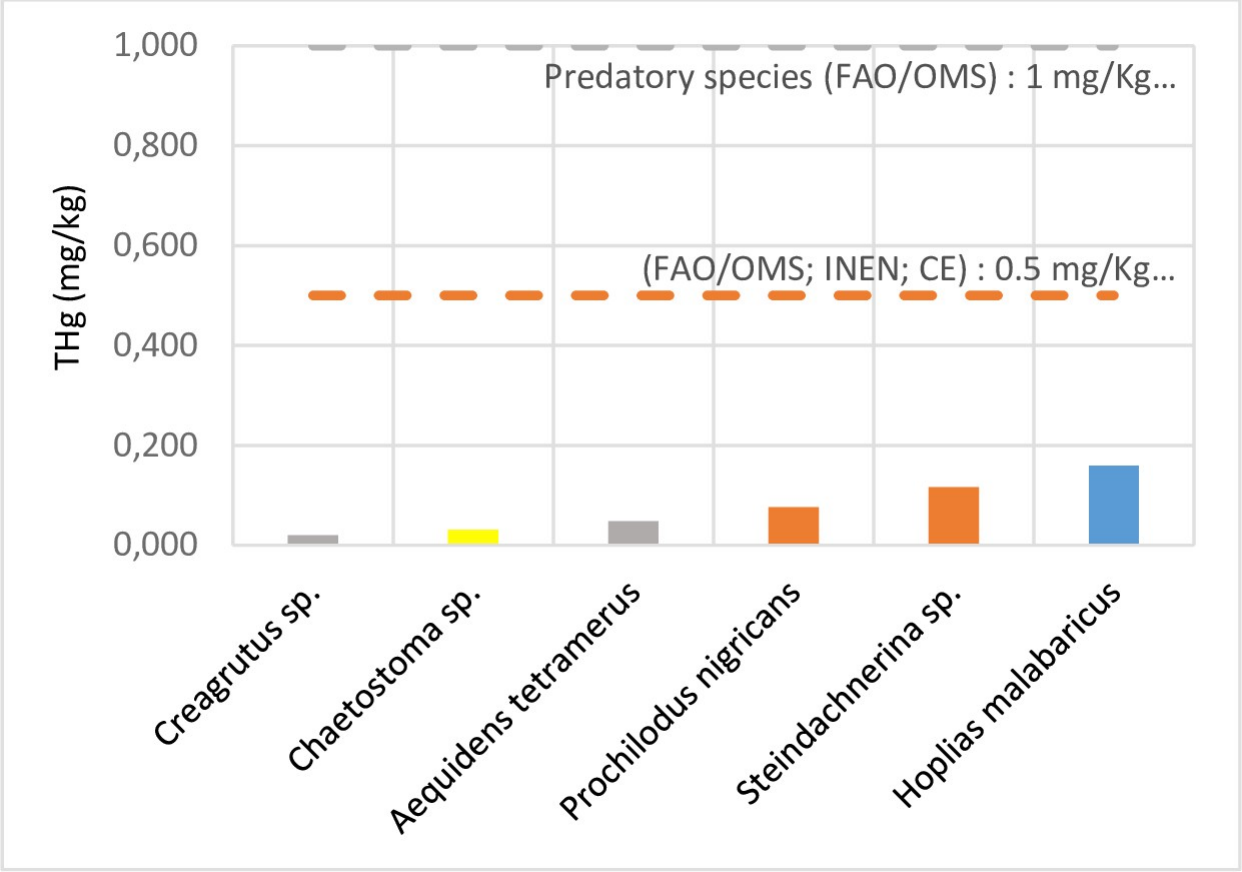
Total mercury concentration (mg kg^-1^) of the species collected in the Pastaza River.

The results for individuals captured at Site 2 are presented in S4 Table. The highest concentration of THg (mg kg^-1^) corresponded to *Hoplias malabaricus* and *Steindachnerina* sp., with mean values of 0.160 ± 0.033 and 0.116 ± 0.031, respectively, while lower levels were obtained for *Creagrutus* sp. and *Chaetostoma* sp., with mean values of 0.020 ± 0.007 and 0.031 ± 0.022, respectively. None of the THg values obtained exceeded the reference limits set by the regulatory bodies. Again, the highest THg concentration was indicated for the carnivorous and detritivorous species, with lower levels in the omnivorous and herbivorous species. The results are summarized in Fig 2.

For individuals from both sites, THg content exhibited the following hierarchy: carnivores > detritivores > omnivores > herbivores, with mean values of 0.115, 0.045, 0.039, and 0.020 mg kg^-1^ (Site 1) (S2A Fig), and 0.160, 0.106, 0.034, and 0.031 mg kg^-1^ (Site 2) (S2B Fig), respectively.

Previous studies have suggested THg biomagnification in organisms at the highest levels of the trophic chain [61, 62]. This idea is consistent with the current study’s results: at both sites, the carnivorous species contained the highest amount of THg. Research on Hg concentration in *Charax* sp. is lacking. However, given the species’ carnivorous nature, the results suggest its potential for higher Hg bioaccumulation compared to omnivorous and herbivorous species [63]. Lancheros Ascencio [64], analyzed the Hg content in muscle tissue of fish species from the Magdalena River in Colombia; the highest Hg concentration was found for the carnivorous species *Roeboides dayi* and *Ageneiosus pardalis*, with values of 1.759 and 1.416 mg kg^-1^, while the herbivorous species *Chaetostoma* sp. and *Hypostomus hondae* had levels of 0.333 and 0.165 mg kg^-1^, respectively. These results are aligned with the THg values obtained in the present study, in which the herbivorous species *Chaetostoma* sp. had the lowest level (0.017 mg kg^-1^). At Site 2, the carnivorous *Hoplias malabaricus* showed the highest THg concentration (0.160 mg kg^-1^). Similar results were reported by Ferreira da Silva *et al*. [20], who monitored Hg levels in fish from the Solimões River in the Amazonian Three Frontiers region (Brazil, Colombia, and Peru) and found Hg levels of 1.465 mg kg^-1^ for *Hoplias malabaricus*, which exceeded the permitted limit (1 mg kg^-1^) for carnivorous fish established by Brazilian regulations. Marrugo-Negrete *et al*. [65] monitored THg concentrations in fish from the Sinú River in Colombia from 2004 to 2009, finding that for the carnivorous-piscivorous *Hoplias malabaricus*, the Hg level (1.39 ± 0.69 mg kg^-1^) exceeded the reference levels for predatory species established by the Codex Alimentarius Commission [59]. They also determined a significant positive correlation (*p* < 0.05) between Hg concentration and fish length, suggesting a process of bioaccumulation within the aquatic food web [65].

The current study assessed differences in THg concentration between individuals from the two sites using nonparametric statistical analysis employing the Mann–Whitney U test at a significance level of 5% (α = 0.05). The analysis yielded a *p* value of 0.4341, indicating no significant differences (*p* > α).

The Kruskal–Wallis test was used to determine significant differences between THg concentrations based on the feeding habits of each individual. Results showed a significant difference in THg values between Site 1 carnivorous species and detritivorous, omnivorous, and herbivorous species (*H* = 192560; *p* = 0.0002; *p* < 0.05). Similarly, for Site 2, significant differences in THg levels were also found according to feeding habits (*H* = 10.4875; *p* = 0.0148; *p* < 0.05).

**Fig 3:**
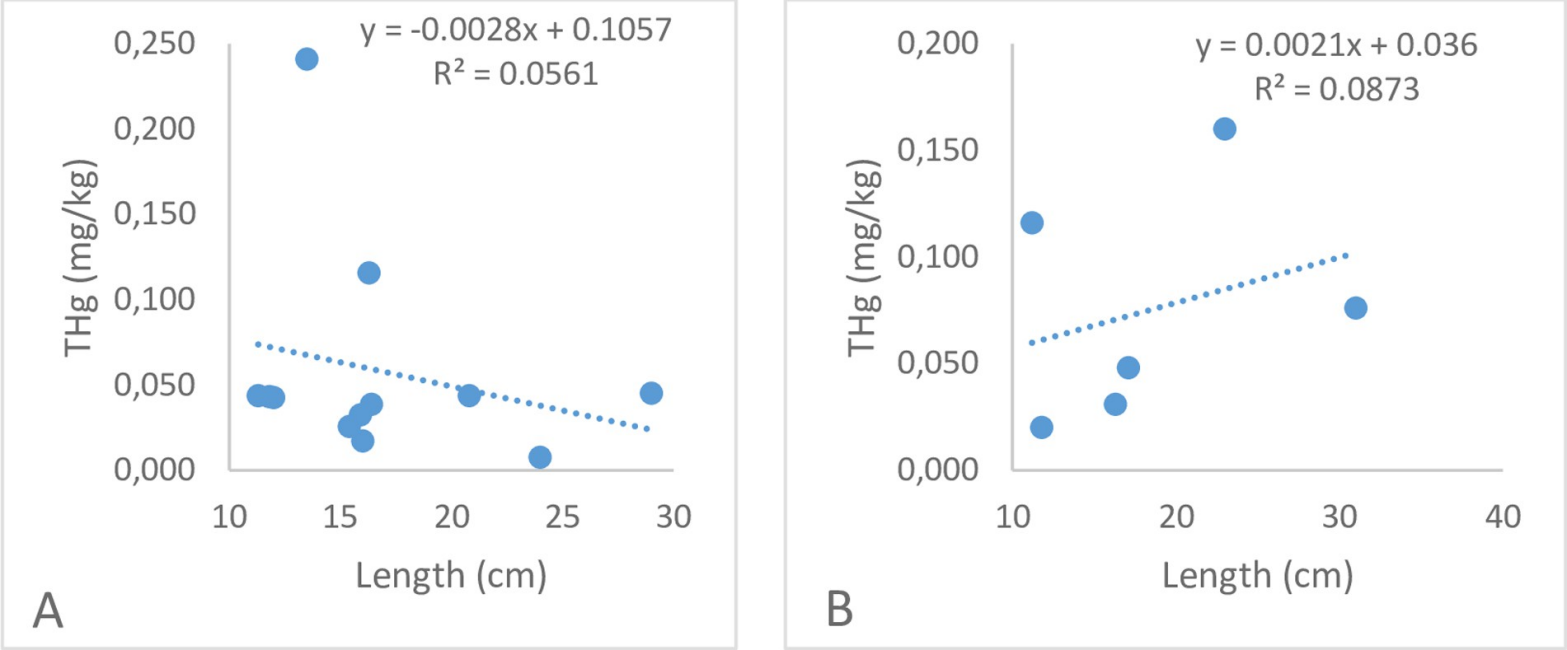
Relationship between mercury concentration and total length of fish species. A) Site 1 and B) Site 2.

Regarding the correlation between THg concentration and species length, Fig 3 indicates no significant correlation (*r* = -0.2368; *p* = 0.4587; *p* > 0.05) for Site 1 individuals, while for Site 2 individuals, there is a positive linear correlation (*r* = 0.2955; *p* = 0.5696; *p* > 0.05).

Exposure time is considered the primary factor contributing to the bioaccumulation of contaminants in fish tissues. Therefore, larger and longer-living fish are expected to accumulate higher levels of metals given their continuous absorption of metals from the environment [22]. The results for Site 2 (*r* = 0.2955; *p* > 0.05) suggest that THg content increased as size increased. This finding is consistent with that reported by Gracia *et al*. [66] who found larger, older fish bioaccumulated higher concentrations of contaminants due to prolonged exposure compared to smaller, younger species. Similarly, Yi and Zhang [11] found that within the same species, the largest individuals had greater Hg levels because of longer exposure time to the pollutant. This type of positive linear pattern can become concerning when fish are heavily consumed by populations living near rivers. In such cases, larger individuals are often preferred for consumption, posing a potential health risk to consumers [67]. In the Pastaza River basin, larger species such as the black prochilodus (*Prochilodus nigricans*), wolf fish (*Hoplias malabaricus*), and saddle cichlid (*Aequidens tetramerus*) are highly valued in subsistence artisanal fishing (S3 Fig). Species such as *Hypostomus* sp. and *Chaetostoma* sp. (S3 Fig) are commonly traded as ornamental fish. Nonetheless, local communities near the rivers utilize them as a source of protein, often incorporating them into soups [43, 68, 69], and is part of the basic diet [70]. The no significant correlation (*r* = -0.2368; *p* > 0.05) between THg concentration and fish length for Site 1 individuals may be because Hg levels may be influenced by geographical area and fish migration patterns [4, 71]. The Pastaza River basin is distinguished by its diverse ecosystems, mainly characterized by alluvial plain forests. These environments are recognized as metals and metalloids accumulation zones because during the floods occurring between December and May, anaerobic bacteria convert inorganic Hg into MeHg [72]. Indeed, studies have documented higher concentrations of contaminants in fish during the high-water season [9]. Additionally, variations in Hg concentrations may also be influenced by the bioavailability of contaminants in the environment. Erosion has been found to affect the presence of Hg in Amazonian ecosystems, as this process transports contaminants from the soil to the rivers [73]. Negative bioaccumulation patterns may seem contradictory to the bioaccumulation process; however, it has been shown that such patterns may be associated with dietary changes that species tend to undergo during their maturation stage [22], as well as species’ migration processes and the availability of the metal in water and sediments [20]. However, factors such us the time of exposure, intake and uptake in the species analyse need to be considered for future studies.

### Human health risk assessment

In Ecuador, fish meat is consumed once or twice per week, however it does not occur every week. By considering a personal portion of 80 g of fish meat in a single serving per week for an adult, and the half for children (40 g), the Rx calculated for the average THg content in the samples is less than 1 for all the fish species analysed. From these results (Table 3) it can be concluded that the consumption of these fish does not represent a non-carcinogenic risk for children and adults. According to the FDA/EPA 2022, the highest allowable THg concentration in fish when eating 1 serving per week is 0.46 mg·kg^-1^. Considering the mean concentrations of MeHg, for all the fish species analysed, the results for daily consumption rate (Rclim) were between 6–199 g and 29–928 g for children and adults, respectively (Table 3). In addition, calculated tHQ values are reported in Table 3. Considering the mean THg concentration in the muscle tissue of the examined fish, all calculated tHQ values were less than 1; result that confirms the consumption of these fish could pose no risk to human health. Although, this Hg concentration is likely to change over time, given the persistent nature of metals in the environment; thus exacerbating the effect [17].

**Table 3:** Human health risk of total mercury content.

Species	THg (mg kg^-1^)	(**)	EDI	tHQ	Ex (mg·kg^-1^ day BW^-1^)	Rx	CR_lim_	CR_lim_x1000
*Chaetostoma* sp.	**0.0170**	Children	6.48E^-05^	1.62E^-04^	6.49E^-06^	0.06493	0.08801	88.006
		Adults	2.75E^-05^	6.88E^-05^	2.78E^-06^	0.02782	0.41069	410.696
*Hypostomus* sp.	**0.0075**	Children	2.87E^-05^	7,17E-05	2.87E^-06^	0.02874	0.19879	198.796
		Adults	1.22E^-05^	3.05E^-05^	1.23E^-06^	0.01231	0.92771	927.717
*Cetopsis plumbea*	**0.1157**	Children	4.40E^-04^	1.10E^-03^	4.41E^-05^	0.44112	0.01295	12.953
		Adults	1.87E^-04^	4.67E^-04^	1.89E^-05^	0.18905	0.06045	60.451
*Cordylancistrus* sp.	**0.0257**	Children	9.77E^-05^	2.44E^-04^	9.79E^-06^	0.09792	0.05835	58.350
		Adults	4.15E^-05^	1.04E^-04^	4.19E^-06^	0.04196	0.27230	272.304
*Pimelodella* sp.	**0.0324**	Children	1.23E^-04^	3.08E^-04^	1.23E^-05^	0.12344	0.04628	46.289
		Adults	5.23E^-05^	1.31E^-04^	5.29E^-06^	0.05290	0.21601	216.018
*Parodon buckleyi*	**0.0387**	Children	1.47E^-04^	3.68E^-04^	1.47E^-05^	0.14752	0.03873	38.733
		Adults	6.25E^-05^	1.56E^-04^	6.32E^-06^	0.06322	0.18075	180.754
*Astyanax bimaculatus*	**0.0430**	Children	1.64E^-04^	4.09E^-04^	1.64E^-05^	0.16397	0.03484	34.847
		Adults	6.95E^-05^	1.74E^-04^	7.03E^-06^	0.07027	0.16262	162.623
*Charax* sp.	**0.2411**	Children	9.16E^-04^	2.29E^-03^	9.19E^-05^	0.91865	0.00622	6.220
		Adults	3.89E^-04^	9.73E^-04^	3.94E^-05^	0.39371	0.02902	29.027
*Steindachnerina* sp.	**0.0437**	Children	1.66E^-04^	4.15E^-04^	1.67E^-05^	0.16652	0.03431	34.314
		Adults	7.06E^-05^	1.76E^-04^	7.14E^-06^	0.07136	0.16013	160.135
*Creagrutus* sp.	**0.04262**	Children	1.62E^-04^	4.05E^-04^	1.62E^-05^	0.16237	0.03519	35.191
		Adults	6.88E^-05^	1.72E^-04^	6.96E^-06^	0.06958	0.16422	164.227
*Prochilodus nigricans*	**0.04501**	Children	1.71E^-04^	4.28E-^04^	1.72E^-05^	0.17171	0.03327	33.278
		Adults	7.28E^-05^	1.82E^-04^	7.36E^-06^	0.07359	0.15529	155.299
*Crenicichla anthurus*	**0.0438**	Children	1.67E^-04^	4.16E^-04^	1.67E^-05^	0.16695	0.03422	34.226
		Adults	7.07E^-05^	1.77E^-04^	7.16E^-06^	0.07155	0.15972	159.725
*Aequidens tetramerus*	**0.0480**		1.83E^-04^	1.83E^-07^	1.83E^-05^	0.18325	0.03118	31.182
		Children	7.77E^-05^	7.77E^-08^	7.85E^-06^	0.07853	0.14551	145.519
Hoplias malabaricus	**0.1600**	Adults	6.09E^-04^	6.09E^-07^	6.10E^-05^	0.61031	0.00936	9.362
		Children	2.59E^-04^	2.59E^-07^	2.62E^-05^	0.26156	0.04369	43.693
*Steindachnerina* sp.	**0.1169**	Adults	4.41E^-04^	4.41E^-07^	4.42E^-05^	0.44223	0.01292	12.921
		Children	1.87E^-04^	1.87E^-07^	1.89E^-05^	0.18953	0.06029	60.299
*Creagrutus* sp.	**0.0200**	Adults	7.70E^-05^	7.70E^-08^	7.72E^-06^	0.07721	0.07400	74.002
		Children	3.27E^-05^	3.27E^-08^	3.31E^-06^	0.03309	0.34534	345.342
*Chaetostoma* sp.	**0.0310**	Adults	1.16E^-04^	1.16E^-07^	1.16E^-05^	0.11620	0.04917	49.173
		Children	4.92E^-05^	4.92E^-08^	4.98E^-06^	0.04980	0.22947	229.474
*Prochilodus nigricans*	**0.0760**	Adults	2.90E^-04^	2.90E^-07^	2.91E^-05^	0.29099	0.01963	19.636
		Children	1.23E^-04^	1.23E^-07^	1.25E^-05^	0.12471	0.09163	91.638

(**) Children (15 kg); Adults (70 kg)

Several case studies at a global level such as Minamata in Japan and Irak [10] document the impacts by intake of metals and metalloids in humans, it has been established that Hg can damage the nervous system; it is also classified as carcinogenic, primarily affecting the liver and esophagus [74]. In pregnant women, Hg can affect the embryo by crossing the placenta, thereby inhibiting fetal brain development. The initial manifestations of Hg poisoning in the human body may include symptoms such as headaches, tremors, fatigue, and lack of coordination of muscle movement [75]. In the municipality of Ayapel in Colombia, the presence of Hg in residents’ hair was linked to the consumption of contaminated fish from the Ayapel marsh, where the concentration exceeded the permissible limit (1 mg kg^-1^); residents showed symptoms including headaches, fatigue, and irritability [66]. A study in Mexico determined that the consumption of Hg-contaminated fish from Lake Chapala contributed to the bioaccumulation of Hg in fetuses; a Hg concentration of 1 mg kg^-1^ was found in the hair of new-borns [76]. Another study in Brazil monitored the effects of Hg on children aged 6–14 years from a riverine population living along the Madeira River whose diet primarily consists of fish. The amount of Hg in the children’s hair ranged from 0.05 to 21.75 mg kg^-1^. These values influenced their neuropsychological functions, with the children showing low scores on IQ assessments [77].

Considering the impact of Hg on human health, it is advisable to reduce the consumption of carnivorous species, as they tend to accumulate higher levels of contaminants in their tissues [78], and instead consume species from lower trophic levels [18]. The ability of fish tissues to accumulate contaminants is related to their metabolic activity. A higher concentration has been observed in hepatic tissue compared to muscular tissue [79], as the liver is metabolically more active, serving as a storage and detoxification site for all substances entering through the bloodstream. Additionally, this is attributed to binding proteins such as metallothioneins [80]. Hence, this capacity to accumulate metals renders the liver the most crucial storage tissue in aquatic species [81]. Conversely, gills are organs capable of accumulating a higher concentration of contaminants compared to muscle tissue. This is attributed to their crucial role in fish physiology, as they are directly exposed to the external environment where they can absorb toxic metals from the water [18]. Thus, it is essential to quantify THg in these organs. Finally, is important to point that [16] find relationship between the consumption of fish that intake mercury by local communities in Napo River valley showing higher fish consumption and hair Hg levels (8.71 and 5.32 lg g^-1^) as compared to an urban community (1.87 lg g^-1^).

To the best of our knowledge, there is limited literature on the implications of consuming Hg-contaminated fish among populations living near the Pastaza River basin. The only prior study was conducted by Echevarría *et al*. [9], who quantified 11 metals in fish muscle tissue, determining that detritivorous species showed elevated levels of Al, Cd, Cu, Mn, Ni, and Zn, herbivorous species had higher concentrations of Al, Cr, Ni, and Zn, and omnivorous species exhibited high levels of As and Fe. In that study, sites were sampled during two field campaigns: in July 2021 (high water season), and in April 2022 to the rising water season. However, precipitation was lower than normal during the 2021, causing abnormally low water levels for that hydrological season and lower than in 2022. Several of the species analyzed coincide with those captured in our study and in particular they report that they did not detect Hg in their samples, a year after that study we have found Hg in the same species. Considering both this report and the current study, it is crucial to conduct further research to assess whether populations living near the Pastaza River basin are affected by consuming contaminated fish. Continuous monitoring of THg and other metals is necessary. Thus, the current study can be considered an important pioneering effort in this regard.

## Conclusions

The current study determined the THg concentration in muscle tissue of fish species from the middle basin of the Pastaza River and become a base line in order to understand the levels and flow or Hg in natural ecosystems. There were no significant differences in THg concentration between species captured in Sites 1 and those from Site 2; however, significant differences (*p* < 0.05) were found when comparing different feeding habits. Regarding the relationship between THg concentration and fish length, there was no significant correlation for the Site 1 species, while a weak positive correlation was observed for Site 2 species. In none of the examined species did THg concentrations exceed the maximum permissible levels established by the technical regulations of INEN, the Codex Alimentarius Commission, and the European Commission. The tHQ indicated that consuming the fish could pose no risk to human health.

## Supporting information

S1 Table Recovery percentages obtained from DORM-4 certified reference material. (PDF)

S2 Table Limits (L) of detection (D) and quantification (Q). (PDF)

S3 Table Total mercury concentration (mg/kg) of the species collected in the Metzeras River (Site1). (PDF)

S4 Table Total mercury concentration (mg/kg) of the species collected in the Pastaza River (Site 2). (PDF)

S1 Fig Middle Pastaza River basin. (PDF)

S2 Fig Diagram of the distribution of THg levels in the different trophic levels. A) Metzeras River and B) Pastaza River.
(PDF)

S3 Fig Individuals identified in: A) Metzeras River and B) Pastaza River.
(PDF)
