# Supplementary material for: Assessment of total mercury content in fish muscle tissue from the middle basin of the Pastaza River, Ecuador
Source: PLoS One. 2024 Dec 18;19(12):e0310688. doi: 10.1371/journal.pone.0310688 (PMC11654945; doi:10.1371/journal.pone.0310688)
Supplement: S1 Table — (PDF) [file pone.0310688.s001.pdf]

S1 Table. Recovery percentages obtained from DORM-4 certified reference material

Sample	Weight (g)	Concentration (μg kg^{-1})	Recovery %
DORM-4	0.0401	379.239	92.05
	0.0401	396.4977	96.24
	0.0406	377.1685	91.55
	0.0406	385.5291	93.58
	0.0406	372.0563	90.30
		**Mean**	**92.74**
		**SD**	**2.28**
		**%CV**	**2.46**
