# Supplementary material for: Assessment of total mercury content in fish muscle tissue from the middle basin of the Pastaza River, Ecuador
Source: PLoS One. 2024 Dec 18;19(12):e0310688. doi: 10.1371/journal.pone.0310688 (PMC11654945; doi:10.1371/journal.pone.0310688)
Supplement: S2 Table — (PDF) [file pone.0310688.s002.pdf]

**S2 Table.** Limits (L) of detection (D) and quantification (Q)

| Sample | Concentration (μg $kg^{-1}$) |
|---|---|
| Background | 1.1581 |
| | 0 |
| | 0 |
| | 0.2083 |
| | 0 |
| | 0 |
| | 0.2223 |
| | 0 |
| | 0 |
| **Mean** | 0.18 |
| **SD** | 0.38 |
| **DL** | 1.14 |
| **QL** | 3.80 |
