# Supplementary material for: Assessment of total mercury content in fish muscle tissue from the middle basin of the Pastaza River, Ecuador
Source: PLoS One. 2024 Dec 18;19(12):e0310688. doi: 10.1371/journal.pone.0310688 (PMC11654945; doi:10.1371/journal.pone.0310688)
Supplement: S3 Table — (PDF) [file pone.0310688.s003.pdf]

S3 Table. Total mercury concentration (mg kg^{-1}) of the species collected in the Metzeras River (Site 1)

Species	n	THg (mg kg^{-1}) ±SD (Dry weight)	THg (mg kg^{-1}) ±SD (Wet weight)
Chaetostoma sp.	2	0.086 ± 0.008	0.017 ± 0.002
Hypostomus sp.	1	0.038± 0.007	0.008± 0.004
Cetopsis plumbea	2	0.610 ± 0.191	0.116 ± 0.045
Cordylancistrus sp.	3	0.132 ± 0.014	0.026 ± 0.004
Pimelodella sp.	3	0.178 ± 0.005	0.032 ± 0.003
Parodon buckleyi	3	0.185 ± 0.138	0.039 ± 0.025
Astyanax bimaculatus	3	0.266 ± 0.047	0.043 ± 0.011
Charax sp.	1	1.352± 0.066	0.241± 0.018
Steindachnerina sp.	1	0.260± 0.073	0.044± 0.031
Creagrutus sp.	1	0.264± 0.087	0.043± 0.056
Prochilodus nigricans	2	0.232 ± 0.094	0.045 ± 0.018
Crenicichla anthurus	3	0.218 ± 0.017	0.044 ± 0.004
