# Supplementary material for: Assessment of total mercury content in fish muscle tissue from the middle basin of the Pastaza River, Ecuador
Source: PLoS One. 2024 Dec 18;19(12):e0310688. doi: 10.1371/journal.pone.0310688 (PMC11654945; doi:10.1371/journal.pone.0310688)
Supplement: S4 Table — (PDF) [file pone.0310688.s004.pdf]

S4 Table: Total mercury concentration (mg kg^{-1}) of the species collected in the Pastaza River (Site 2)

Species	n	THg (mg kg^{-1}) ±SD (Dry weight)	THg (mg kg^{-1}) ±SD (Wet weight)
Aequidens tetramerus	3	0.255±0.018	0.048±0.003
Hoplias malabaricus	2	0.834±0.237	0.160±0.033
Steindachnerina sp.	3	0.596±0.135	0.116±0.031
Creagrutus sp.	3	0.131±0.021	0.020±0.007
Chaetostoma sp.	3	0.165±0.112	0.031±0.022
Prochilodus nigricans	1	0.290±0.116	0.076±0.003
