# Supplementary figures and images for: Assessment of total mercury content in fish muscle tissue from the middle basin of the Pastaza River, Ecuador
Source: PLoS One. 2024 Dec 18;19(12):e0310688. doi: 10.1371/journal.pone.0310688 (PMC11654945; doi:10.1371/journal.pone.0310688)

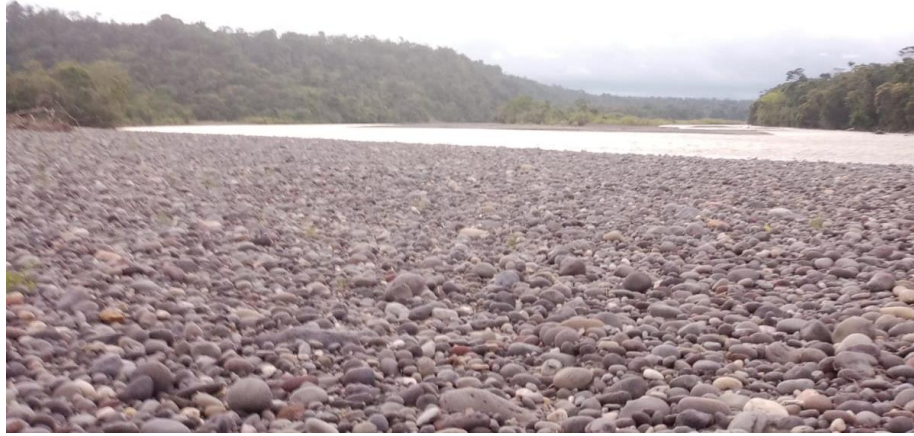

Supplement: S1 Fig — (PDF) [file pone.0310688.s005.pdf]

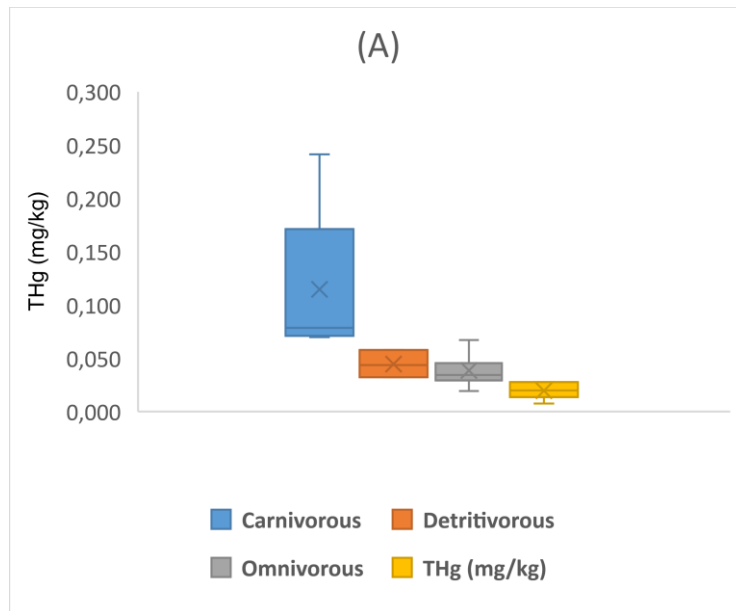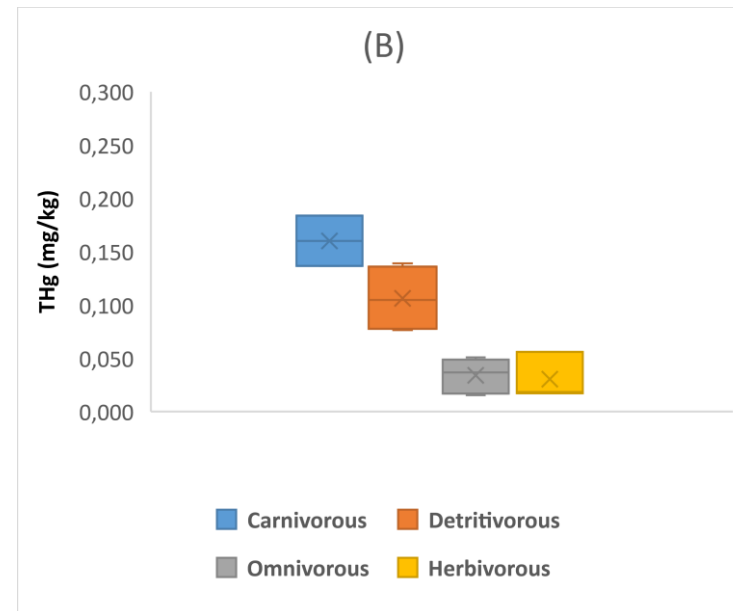

Supplement: S2 Fig — A) Metzeras River and B) Pastaza River. (PDF) [file pone.0310688.s006.pdf]
