# Supplementary material for: Assessment of total mercury content in fish muscle tissue from the middle basin of the Pastaza River, Ecuador
Source: PLoS One. 2024 Dec 18;19(12):e0310688. doi: 10.1371/journal.pone.0310688 (PMC11654945; doi:10.1371/journal.pone.0310688)
Supplement: S3 Fig — Individuals identified in: A) Metzeras River and B) Pastaza River. (PDF) [file pone.0310688.s007.pdf]

<table>
<tr><th colspan="2">A)</th></tr>
<tr><td>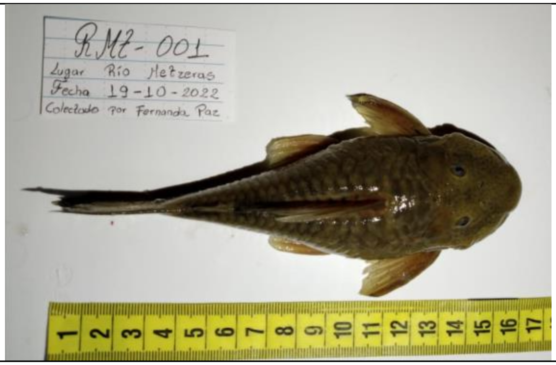
</td><td>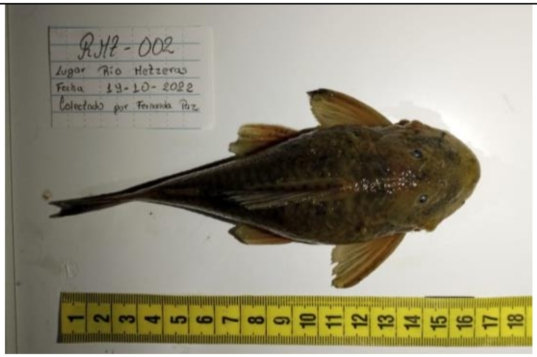
</td></tr>
<tr><td>Order: Siluriformes
Family: Loricariidae
Gender: Chaetostoma</td><td>Order: Siluriformes
Family: Loricariidae
Gender: Chaetostoma</td></tr>
<tr><td>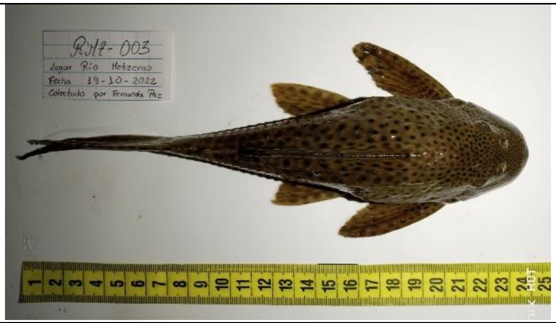
</td><td>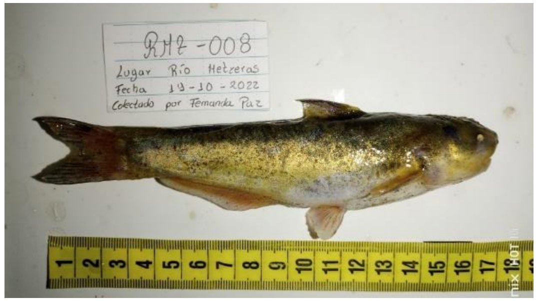
</td></tr>
<tr><td>Order: Siluriformes
Gender: Loricariidae
Species: Hypostomus</td><td>Order: Siluriformes
Family: Cetopsidae
Gender: Cetopsis
Species: Cetopsis plumbea</td></tr>
</table>

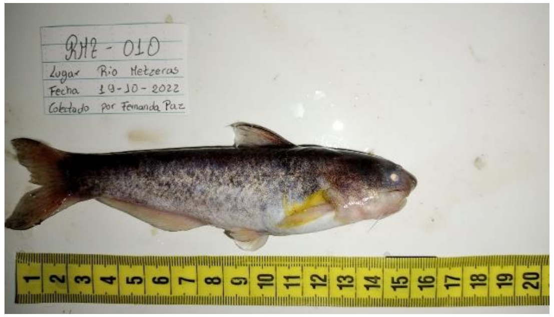	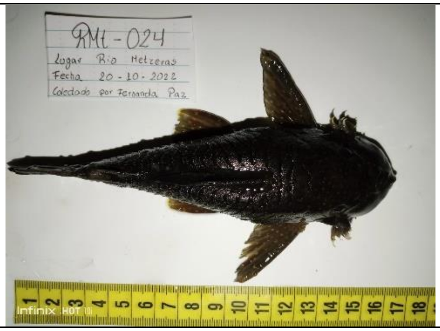
**Order:** Siluriformes<br>**Family:** Cetopsidae<br>**Gender:** Cetopsis<br>**Species:** Cetopsis plumbea	**Order:** Siluriformes<br>**Family:** Loricariidae<br>**Gender:** Cordylancistrus
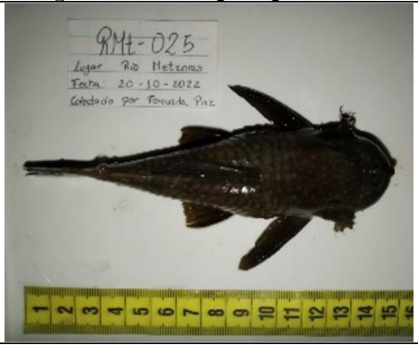	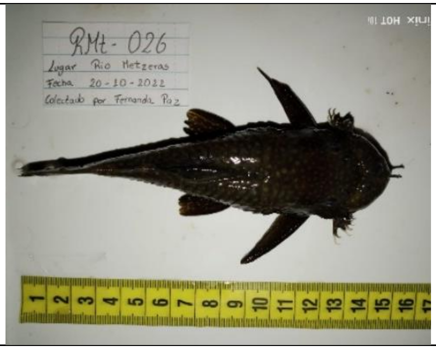
**Order:** Siluriformes<br>**Family:** Loricariidae<br>**Gender:** Cordylancistrus	**Order:** Siluriformes<br>**Family:** Loricariidae<br>**Gender:** Cordylancistrus

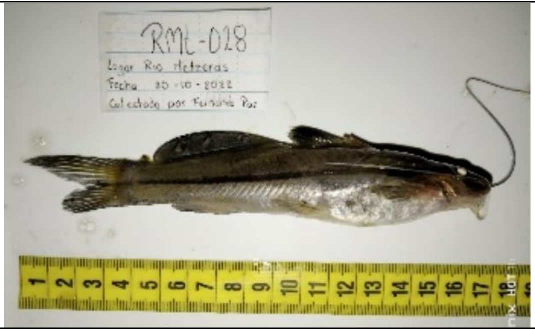	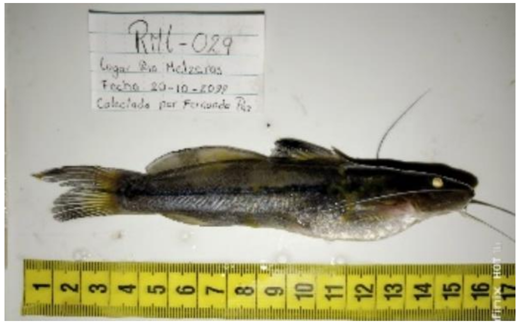
**Order:** Siluriformes<br>**Family:** Heptapteridae<br>**Gender:** Pimelodella	**Order:** Siluriformes<br>**Family:** Heptapteridae<br>**Gender:** Pimelodella
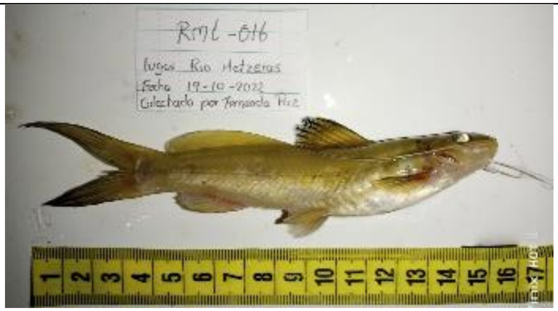	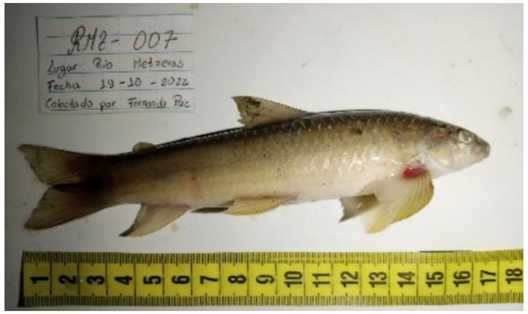
**Order:** Siluriformes<br>**Gender:** Heptapteridae<br>**Species:** Pimelodella	**Order:** Characiformes<br>**Family:** Parodontidae<br>**Gender:** Parodon<br>**Species:** Parodon buckleyi

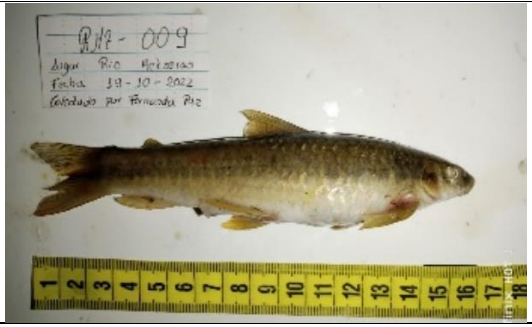

Order: Characiformes
Family: Parodontidae
Gender: Parodon
Species: Parodon buckleyi

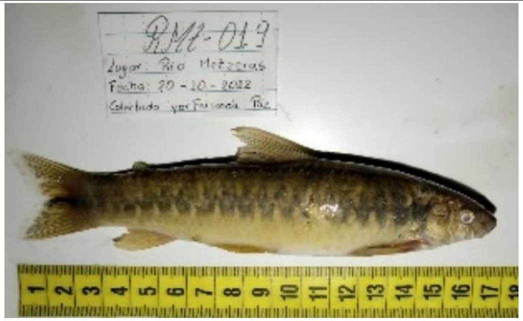

Order: Characiformes
Family: Parodontidae
Gender: Parodon
Species: Parodon buckleyi

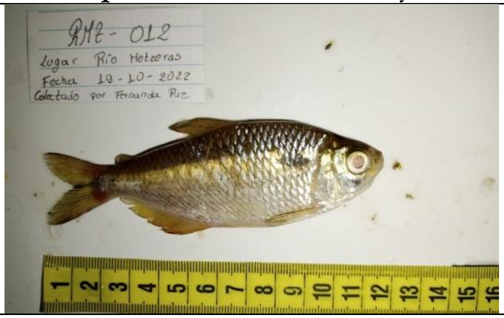

Order: Characiformes
Family: Characidae
Gender: Astyanax
Species: Astyanax bimaculatus

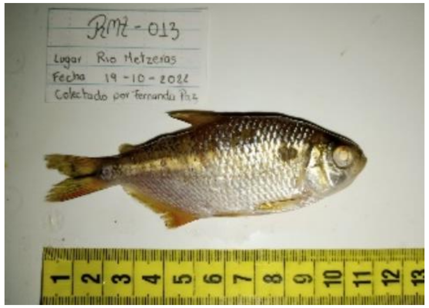

Order: Characiformes
Family: Characidae
Gender: Astyanax
Species: Astyanax bimaculatus

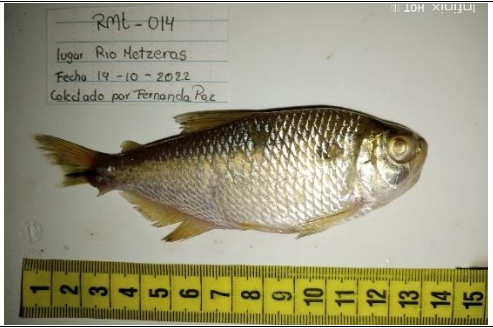	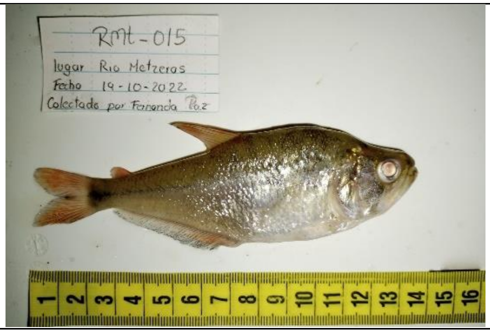
**Order:** Characiformes<br>**Family:** Characidae<br>**Gender:** Astyanax<br>**Species:** Astyanax bimaculatus	**Order:** Characiformes<br>**Gender:** Characidae<br>**Species:** Charax
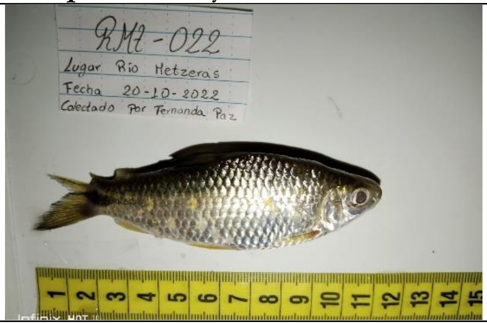	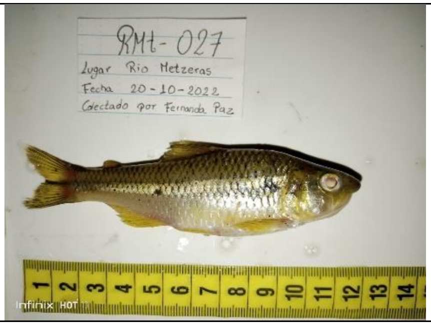
**Order:** Characiformes<br>**Gender:** Curimatidae<br>**Species:** Steindachnerina	**Order:** Characiformes<br>**Gender:** Characidae<br>**Species:** Creagrutus

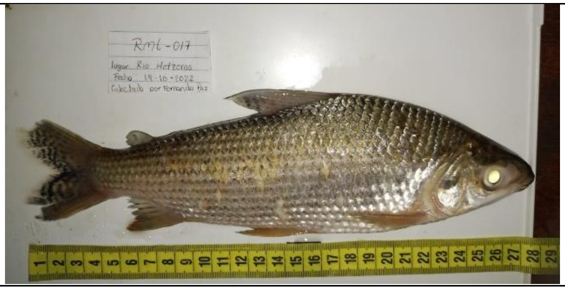	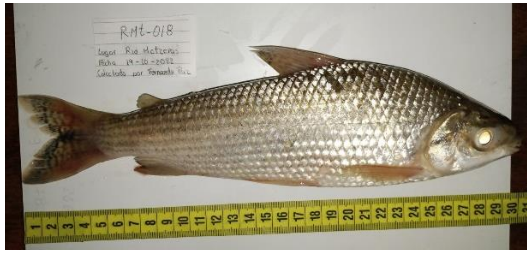
**Order:** Characiformes<br>**Family:** Prochilodontidae<br>**Gender:** Prochilodus<br>**Species:** Prochilodus nigricans	**Order:** Characiformes<br>**Family:** Prochilodontidae<br>**Gender:** Prochilodus<br>**Species:** Prochilodus nigricans
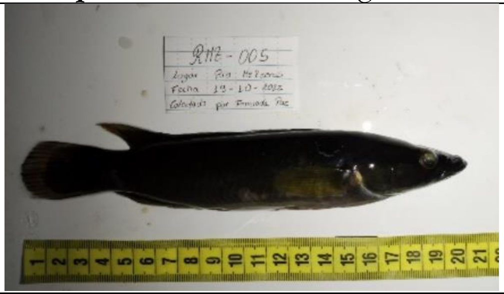	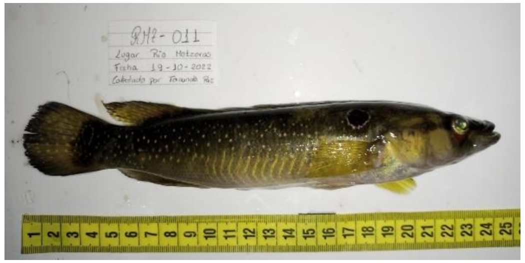
**Order:** Cichliformes<br>**Family:** Cichlidae<br>**Gender:** Crenicichla<br>**Species:** Crenicichla anthurus	**Order:** Cichliformes<br>**Family:** Cichlidae<br>**Gender:** Crenicichla<br>**Species:** Crenicichla anthurus

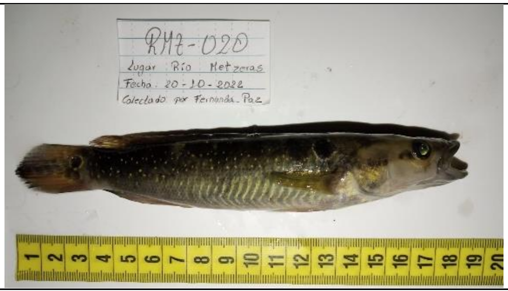	
**Order:** Cichliformes<br>**Family:** Cichlidae<br>**Gender:** Crenicichla<br>**Species:** Crenicichla anthurus	

**B)**	
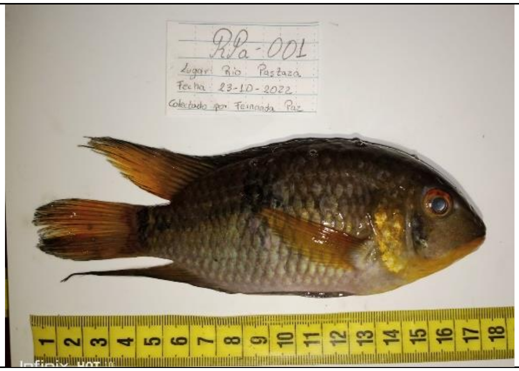	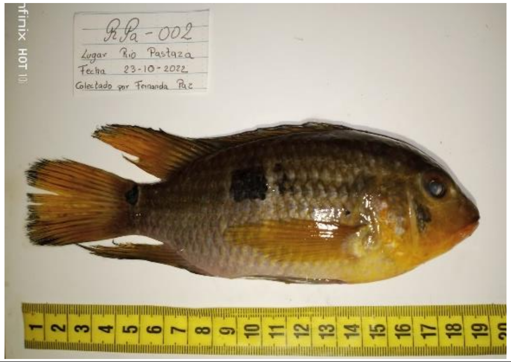
**Order:** Cichliformes<br>**Family:** Cichlidae<br>**Gender:** Aequidens<br>**Species:** Aequidens tetramerus	**Order:** Cichliformes<br>**Family:** Cichlidae<br>**Gender:** Aequidens<br>**Species:** Aequidens tetramerus

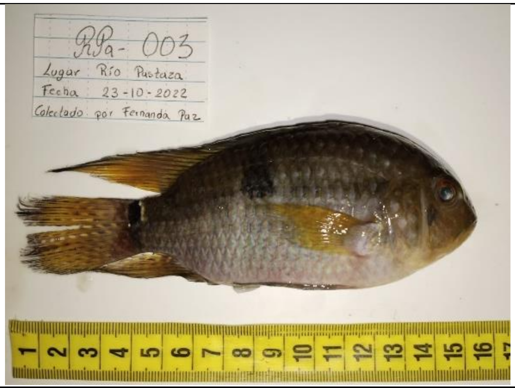	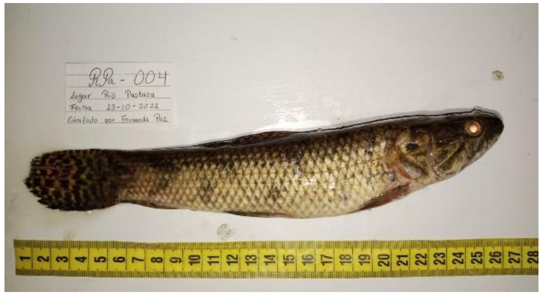
**Order:** Cichliformes<br>**Family:** Cichlidae<br>**Gender:** Aequidens<br>**Species:** Aequidens tetramerus	**Order:** Characiformes<br>**Family:** Erythrinidae<br>**Gender:** Hoplias<br>**Species:** Hoplias malabaricus
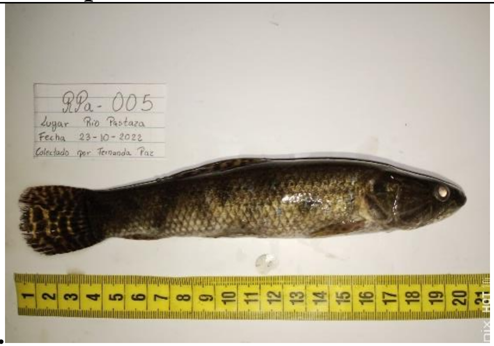	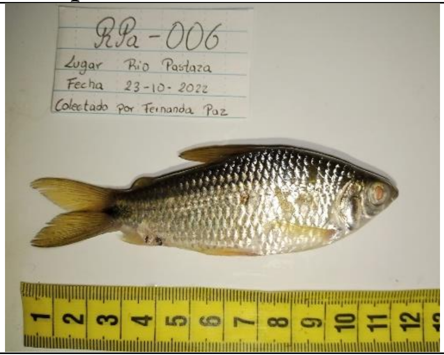
**Order:** Characiformes<br>**Family:** Erythrinidae<br>**Gender:** Hoplias<br>**Species:** Hoplias malabaricus	**Order:** Characiformes<br>**Family:** Curimatidae<br>**Gender:** Steindachnerina

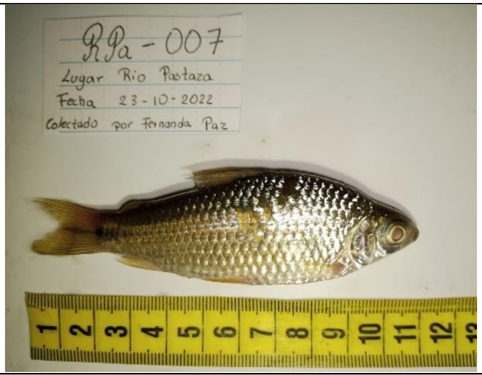	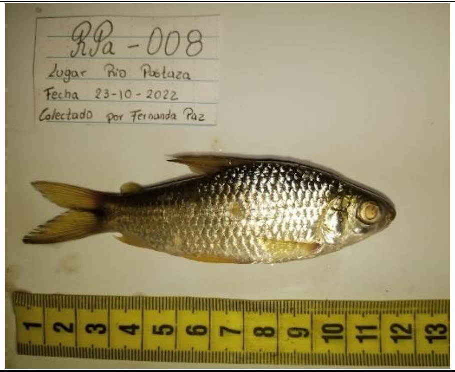
**Order:** Characiformes<br>**Family:** Curimatidae<br>**Gender:** Steindachnerina	**Order:** Characiformes<br>**Family:** Curimatidae<br>**Gender:** Steindachnerina
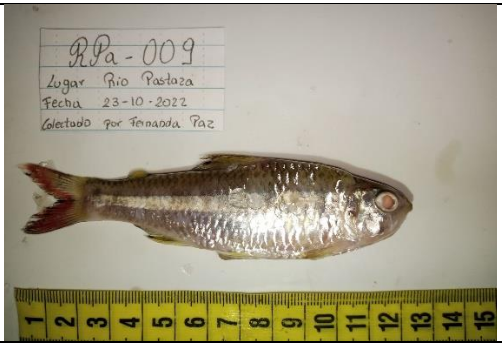	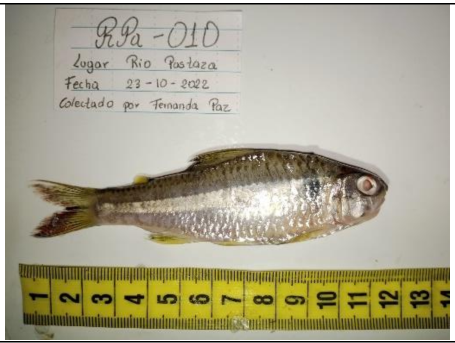
**Order:** Characiformes<br>**Family:** Characidae<br>**Gender:** Creagrutus	**Order:** Characiformes<br>**Family:** Characidae<br>**Gender:** Creagrutus

<table>
<tr><td>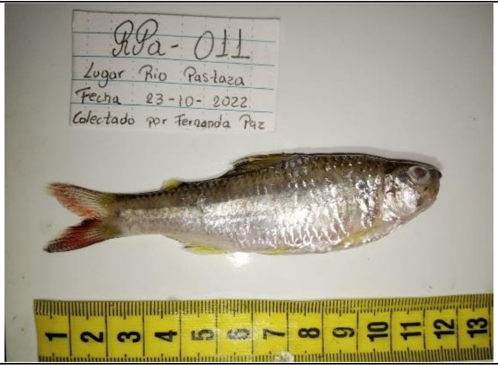
</td><td>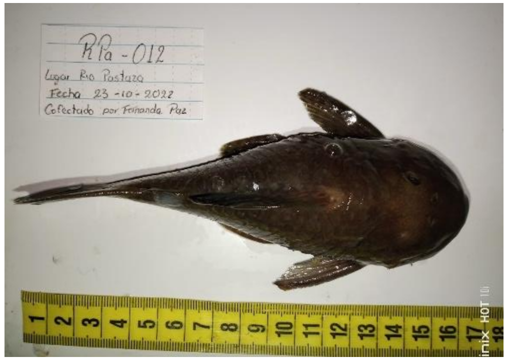
</td></tr>
<tr><td>Order: Characiformes
Family: Characidae
Gender: Creagrutus</td><td>Order: Siluriformes
Family: Loricariidae
Gender: Chaetostoma</td></tr>
<tr><td>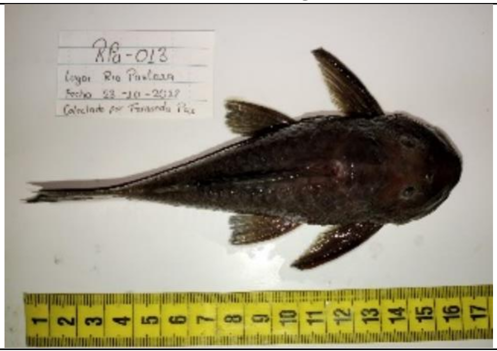
</td><td>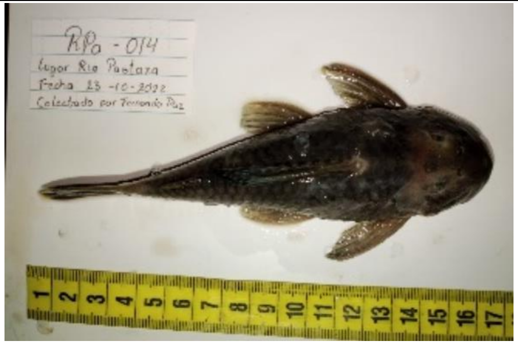
</td></tr>
<tr><td>Order: Siluriformes
Family: Loricariidae
Gender: Chaetostoma</td><td>Order: Siluriformes
Family: Loricariidae
Gender: Chaetostoma</td></tr>
</table>

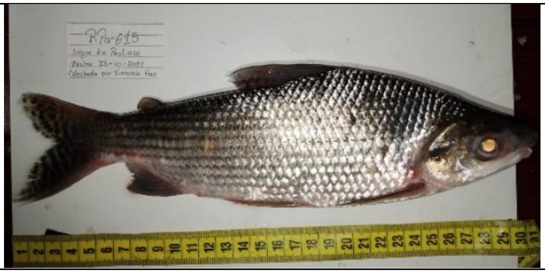	
**Order:** Characiformes<br>**Family:** Prochilodontidae<br>**Gender:** Prochilodus<br>**Species:** Prochilodus nigricans	
